# Phase Separation Drives the Spatial Distribution of Waxes in Agar/Maltodextrin Emulsified Films: Relationships Between Structure and Performance

**DOI:** 10.3390/foods15193459

**Published:** 2026-09-28

**Authors:** Rui Zhang, Yue Sun, Wentao Wang, Hanxue Hou

**Affiliations:** 1 Shandong Key Laboratory of Applied Technology for Protein and Peptide Drugs, School of Pharmaceutical Sciences and Food Engineering, Liaocheng University, Liaocheng 252000, China; 2 Engineering and Technology Center for Grain Processing of Shandong Province, College of Food Science and Engineering, Shandong Agricultural University, Tai’an 271018, China; 3 Department of Food Science and Engineering, Shandong Agriculture and Engineering University, Jinan 250000, China

**Keywords:** waxes, agar/maltodextrin, phase separation, pseudo-bilayer film, spatial distribution

## Abstract

Phase separation-induced bilayer construction offers a one-step strategy to reduce the moisture susceptibility of polysaccharide edible films, yet the spatial distribution mechanism of the hydrophobic phase remains unclear. Herein, agar/maltodextrin (AM) films incorporated with five distinct waxes (soy wax, paraffin wax, beeswax, candelilla wax, and carnauba wax) were fabricated to investigate how the intrinsic melting behaviors of lipids regulate phase separation, microstructural evolution, and film properties. ATR-FTIR analysis indicated that the upward migration and aggregation of waxes weakened the molecular interactions within the upper AM matrix. SEM and AFM observations confirmed that the formation of a wax-enriched surface layer is strongly dependent on the relationship between the wax melting point and the film-drying temperature. The AM-candelilla wax (AM-CW) film exhibited the most distinct phase separation, forming a pseudo-bilayer structure that yielded the lowest water vapor permeability (3.82 × 10^−13^ g·m^−1^·s^−1^·Pa^−1^). Conversely, carnauba wax (CRW), possessing a melting point higher than the drying temperature, underwent premature crystallization before the matrix fully dried. This hindered full stratification and resulted in a random wax distribution within the AM network, compromising the moisture barrier. Furthermore, DSC, XRD, and Raman spectroscopy showed that higher wax melting temperatures induced greater crystalline ordering. This highly ordered structure provided the AM-CRW film with the highest tensile strength (22.57 MPa) and Young’s modulus (867.99 MPa), but at the expense of a significantly reduced elongation at break (3.59%). These findings demonstrate that matching lipid melting properties with processing temperatures is critical for tailoring phase separation and optimizing the barrier performance of biopolymer-based food packaging.

## 1. Introduction

Traditional petroleum-derived plastics continue to dominate global food packaging due to their exceptional physical barrier properties and industrial cost-effectiveness. However, the overuse of non-degradable plastic packaging exacerbates the ecological crisis and associated microplastic health risks [1,2,3]. This trend has profoundly accelerated the development of biodegradable, edible, and sustainable alternatives [4]. Within the realm of renewable biopolymers, polysaccharides are increasingly used in biodegradable food packaging owing to their natural, renewable, and non-toxic properties [5]. Agar, a plant-derived polysaccharide extracted from red algae (*Gelidium* and *Gracilaria* species), exhibits effective film-forming properties and high compatibility with other biopolymers [6]. Despite these advantages, pure agar films are inherently brittle and possess poor barrier properties, limiting their utilization as a film matrix [7,8]. To mitigate these structural drawbacks, maltodextrin, obtained via starch hydrolysis, is integrated into the film matrix as a plasticizing agent, reducing brittleness while enhancing film flexibility [9]. Previous studies show that agar forms a continuous network, whereas maltodextrin acts as a filler within this matrix [10]. Although the mechanical properties are improved, the resulting polysaccharide-based films still exhibit weak water barrier properties. This limitation primarily stems from their intrinsic hydrophilic nature [11,12], further exacerbated by the high hygroscopicity and solubility contributed by maltodextrin [10], which restricts their practical applications in food packaging, particularly for food products with high moisture content.

To address this key limitation, integrating hydrophobic lipids into the hydrophilic biopolymer matrix represents an effective modification strategy. Waxes exhibit superior water barrier properties compared to other lipids due to their abundant fatty alcohols and long-chain alkanes [13]. The wax domains within the film can decrease water absorption, enhance the effective hydrophobic proportion, and reconfigure microstructural pathways [14]. Numerous studies have verified the ability of waxes to improve the water barrier properties of polysaccharide-based films. For instance, studies have demonstrated that the incorporation of natural waxes, such as beeswax, carnauba wax, and candelilla wax, can significantly enhance the surface hydrophobicity and water resistance of composite films based on cassava starch, konjac glucomannan, and pullulan [15,16,17]. As a hydrophobic additive, the intrinsic melting point of the wax is a key parameter that influences the final properties of the films. Different waxes markedly affect physical characteristics such as melting behavior, mechanical strength, and hydrophobicity; specifically, the melting point governs the particle size and morphology within the matrix during the film-forming process [18,19].

The fabrication of polysaccharide-wax composite films has relied on two primary approaches: coating/lamination techniques and emulsification methods. While conventional bilayer films produced via coating/lamination techniques can provide better water resistance, their preparation involves tedious, multi-step casting and drying cycles [20]. Furthermore, due to the distinct incompatibility between the hydrophilic polysaccharide matrix and the hydrophobic lipid coating, such bilayer films tend to delaminate over time and suffer from structural cohesion defects such as cracks, pinholes, and uneven surfaces [21]. Although the emulsification method simplifies preparation, the discontinuous lipid dispersion within the polysaccharide matrix fails to form an effective moisture barrier, rendering its hydrophobic efficiency inferior to that of coating or lamination techniques. Crucially, in emulsion systems, lipid is not always uniformly distributed because emulsions are thermodynamically unstable and prone to breakdown mechanisms like flocculation, particle coalescence, gravitational separation, and phase separation [22]. These phenomena drive lipid droplets to migrate and aggregate, creating localized areas or floating surface layers with high wax content. The intensity of this movement and the final distribution depend heavily on emulsion stability, lipid concentration, droplet size, continuous phase viscosity and the properties of the interfacial surface of wax droplets [23]. Understanding the exact wax distribution is fundamentally critical for modulating the film’s structure and performance.

Consequently, the conception of a self-assembling pseudo-bilayer structure has been proposed, driven by buoyancy-induced wax upward migration and phase separation in thermodynamically unstable emulsions during the film formation process [24,25]. The dispersed lipid droplets coalesce at the air-liquid interface, thereby spontaneously forming a wax-enriched hydrophobic surface layer that remains structurally above a dense, continuous polysaccharide matrix. This structure may effectively combine the barrier advantages of a bilayer with the simplicity of an emulsion film. Moreover, our previous research established the preparation conditions for fabricating agar/maltodextrin-beeswax pseudo-bilayer films, confirming their excellent water vapor barrier properties [24]. Nevertheless, the systematic effects of different waxes with varying melting temperatures on phase separation in such pseudo-bilayer films remain unclear.

Therefore, we hypothesize that the distinct thermal properties of different waxes will dictate their phase separation behavior, thereby governing their ultimate spatial distribution, crystallization behavior, and intermolecular interactions within the continuous matrix. To systematically address this hypothesis, the present study investigates the spatial and structural evolution of agar/maltodextrin (AM) pseudo-bilayer films incorporated with five distinct commercial waxes possessing varying melting behavior, including soy wax (SW, ~50 °C), paraffin wax (PW, ~60 °C), beeswax (BW, ~64 °C), candelilla wax (CW, ~70 °C), and carnauba wax (CRW, ~80 °C). By systematically characterizing the degree of phase separation, interfacial micromorphology, crystallization behavior, and physicochemical properties of the resulting films, this work aims to elucidate how the spatial distribution of diverse waxes fundamentally regulates the structure–performance relationships of polysaccharide-based packaging.

## 2. Materials and Methods

### 2.1. Materials

Food-grade agar (ash content ≤ 1.5%, gel strength: 800–1000 g/cm^2^) was sourced from Yinuo Biological Technology Co., Ltd. (Jinhua, China). Maltodextrin (dextrose equivalent: 18, sulphated ash ≤ 0.5%) was supplied by Shandong Xi’wang Food Co., Ltd. (Binzhou, China). Soy wax (SW, melting point: 48–52 °C) was procured from Mulan Wax Industry Co., Ltd. (Cangzhou, China). Food-grade paraffin wax (melting point: 58–62 °C) was provided by Weijia Industry Co., Ltd. (Jingmen, China). Beeswax (melting point: 62–67 °C) was obtained from Purui Development Co., Ltd. (Zhengzhou, China). Candelilla wax (CW, melting point: 68–72 °C) and carnauba wax (CRW, melting point: 80–86 °C) were purchased from Likangweiye Technology Co., Ltd. (Beijing, China). Analytical-grade glycerol was sourced from Kaitong Chemical Reagent Co., Ltd. (Tianjin, China).

### 2.2. Film Preparation

Equal masses of agar and maltodextrin powders were individually dispersed in deionized water. Each suspension was heated at 95 °C with a water bath (HH-2, Youlian, Changzhou, China) under continuous mechanical stirring (JJ-1, Youlian, Changzhou, China) at 450 rpm for 1 h and subsequently mixed to form a 2% (*w*/*v*) blended AM solution. Glycerol was then added to the mixture at a concentration of 0.60% (*w*/*v*), accounting for 30% of the total AM mass. Subsequently, SW, PW, BW, CW, and CRW were separately introduced into the resulting solutions, also at 0.60% (*w*/*v*), corresponding to 30% of the AM mass. Each solution was stirred under the same conditions (95 °C, 450 rpm) for 30 min and then homogenized using an IKA Ultra Turrax T18 digital homogenizer (T18, IKA, Staufen, Germany) at 8000 rpm for 1 min. The final emulsions were cast into circular dishes and then dried in a constant temperature and humidity chamber (CZ-A-150C, Zhongzhi, Dongguan, China) maintained at 80 °C and 50% relative humidity. Through the above procedures, pseudo-bilayer films of agar/maltodextrin-wax (AM-wax) were successfully fabricated. The schematic representation of the film preparation process is illustrated in Figure 1.

### 2.3. Scanning Electron Microscopy (SEM)

To prepare the samples for cross-sectional imaging, the films were rapidly frozen in liquid nitrogen. After freezing, they were fractured into small chips. Both surface and cross-sectional features were examined using a scanning electron microscope (Quanta FEG 250, FEI, Hillsboro, OR, USA) operating at an acceleration voltage of 5.0 kV. Prior to imaging, the specimens were mounted on a bronze stub and coated with a gold layer to enhance conductivity.

### 2.4. Atomic Force Microscopy (AFM)

Atomic force microscopy (Dimension Icon, Bruker, Karlsruhe, Germany) was used to examine the surface texture of the films. All images were acquired in tapping mode across a 10 μm × 10 μm scan area, with a resolution of 256 × 256 pixels and a scan rate of 1 Hz.

### 2.5. Attenuated Total Reflectance-Fourier Transform Infrared (ATR-FTIR) Spectroscopy

ATR-FTIR spectra were acquired on a Nicolet iS5 spectrometer equipped with an iD5 ATR accessory (Thermo Fisher Scientific, Waltham, MA, USA). Measurements were taken at a resolution of 4 cm^−1^, accumulating 32 scans in the range of 400–4000 cm^−1^. Spectral processing, including baseline correction, was carried out using Omnic 8 (Thermo Fisher Scientific, Waltham, MA, USA) and OriginPro 2017 (OriginLab, Northampton, MA, USA) software.

### 2.6. X-Ray Diffraction (XRD) Analysis

XRD patterns were collected on a D8 Advance diffractometer (Bruker-AXS, Karlsruhe, Germany) using a Cu target (λ = 0.15406 nm) at 40 kV and 30 mA. Scans were performed over a diffraction angle (2θ) range of 5° to 40° with a scan rate of 0.02°/s. The relative crystallinity (%) was determined based on the ratio of the crystalline peak area to the total diffraction area, as calculated using MDI Jade 6 software (Materials Data Inc., Livermore, CA, USA).

### 2.7. Differential Scanning Calorimetry (DSC)

Thermal properties of the films were evaluated using a DSC 200PC instrument (NETZSCH, Selb, Germany). Samples weighing approximately 5 mg were sealed in aluminum crucibles, with an empty crucible serving as the reference. The heating temperature ranged from 0 to 200 °C at a rate of 10 °C/min. Melting temperature (T_m_) and enthalpy change (ΔH) were recorded to characterize the thermal behavior during melting.

### 2.8. Mechanical Properties

Tensile strength (TS), elongation at break (EAB), and Young’s modulus (YM) were determined with an XLW (PC) automated tensile tester (Labthink Instruments, Jinan, China). Mechanical properties were determined according to the standard ASTM D882 method [26]. Film specimens were cut into strips measuring 150 × 15 mm. At least six replicates were measured per sample. Tests were conducted at a testing speed of 100 mm/min and an initial clamp distance of 100 mm. Thickness was recorded at six random points using a micrometer (211-101F, Guanglu, Guilin, China). YM was defined as the ratio of tensile stress to tensile strain within the proportional elastic limit.

### 2.9. Water Vapor Permeability (WVP)

WVP was measured using a PERME W3/030 tester (Labthink Instruments, Jinan, China) according to the GB/T 1037-2021 standard [27]. Circular samples (80 mm diameter) were conditioned at 38 °C and 90% RH after a 4 h pre-equilibration. Weight changes were recorded every 2 h. Three replicates were tested per film sample. Lower WVP values indicate better water vapor barrier performance.

### 2.10. Raman Spectroscopy

Raman spectral data for the film samples were acquired on a DXR2xi laser Raman imaging system (Thermo Fisher Scientific, Waltham, MA, USA). Each spectrum was acquired over 35 scans with a spectral resolution of 8 cm^−1^, at a scanning speed of 100 Hz, across the wavenumber range of 50–3500 cm^−1^.

### 2.11. Light Transmittance

Film strips (4 cm × 1 cm) were analyzed using a UV-8000A spectrophotometer (Metash Instruments, Shanghai, China) from 400–800 nm.

### 2.12. Dynamic Contact Angle (DCA) and Surface Energy

Dynamic contact angles were measured with a contact angle goniometer (JC-2000C1, Zhongchen, Shanghai, China). A 7 μL deionized water droplet was placed on the film surface. A high-speed charge-coupled device (CCD) camera captured images at 0.3 s intervals over a 150 s period to track dynamic contact angle changes. The obtained contact angle data were subsequently processed through drop shape analysis software.

Surface energy evaluation was performed using a JC-2000C1 contact angle goniometer with the two-liquid method. Water and diiodomethane served as the test liquids for these measurements. Water exhibits dispersive and polar components of 21.8 mN/m and 51.0 mN/m, respectively, while diiodomethane shows corresponding values of 48.5 mN/m (dispersive) and 2.3 mN/m (polar). Contact angles for each liquid were determined from captured images using the instrument’s software. Surface energy and its components were then calculated through Wu’s harmonic mean method [28] implemented within the same software platform.

### 2.13. Statistical Analysis

Statistical significance was evaluated using ANOVA in SPSS 21 (IBM Co., New York, NY, USA). All data are presented as the mean ± standard deviation. Data were compared using Duncan’s multiple range test at a significance level of *p* < 0.05.

## 3. Results and Discussion

### 3.1. Microstructure

The SEM images of the surfaces and cross-sections of AM-wax composite films are presented in Figure 2I, while the schematic diagrams of wax spatial distribution in film-forming solution and film cross-sectional structure are shown in Figure 3. All films exhibited irregular, rough surfaces due to the migration of waxes towards the upper layer. Films containing waxes with different melting points displayed markedly different surface structures and varying degrees of phase separation. Fundamentally, the spatial distribution of wax is dictated by the destabilization of the film-forming emulsion during the drying process. Driven by thermodynamic interfacial incompatibility and density differences between the lipid and aqueous phases, the dispersed wax droplets undergo continuous upward migration and coalescence. For waxes with relatively lower melting points (SW to CW), the droplets have sufficient mobility to coalesce and form a continuous layer before the AM matrix fully solidifies, resulting in a distinct phase-separated layered structure (an upper wax-rich layer and a dense underlying AM network). This AM network matches the SEM morphology of the AM film from our prior work [29]. This phase separation minimizes the presence of hydrophobic wax droplets within the underlying hydrophilic phase. The reduction in dispersed wax droplets alleviates physical obstruction, allowing the agar and maltodextrin molecules to interact closely via hydrogen bonding and maintain a continuous and compact network beneath the wax layer.

In contrast, the AM-CRW film (Figure 2(E_3_)) displayed incomplete stratification, with CRW particles (indicated by arrows) remaining embedded within the AM network. This behavior can be ascribed to the high melting point of CRW, which causes it to rapidly crystallize and solidify during the film-forming process. The solidified CRW particles exhibit limited mobility and are prematurely trapped by the progressively solidifying AM network. Compared to the phase-separated structure where the AM matrix remains highly continuous beneath the wax layer, this dispersion of incompletely coalesced, rigid CRW domains physically interrupts the polymer chains. As shown in Figure 3, agar molecules, which adopted random coil conformations in the film-forming solution, gradually assembled into a partial helical structure and formed an AM matrix network as moisture progressively evaporated during drying. The formation of this AM network further inhibited the upward migration of CRW. Dispersed lipid droplets act as structural discontinuities, resulting in a heterogeneous polymer network [21].

However, both the surface and cross-sectional images of the AM-SW film (Figure 2(A_1–3_)) exhibited noticeable void structures (indicated by arrows), suggesting that the wax layer formed by SW was comparatively loose. This phenomenon may be attributed to the inadequate crystallization of SW [30]. This finding is further corroborated by XRD and Raman spectroscopy results (Section 3.5 and Section 3.6). The cross-section of the AM-BW film (Figure 2(C_3_)) showed partial cracking (indicated by arrows) at the interface between the wax layer and the underlying AM matrix, likely resulting from the limited compatibility between BW and the AM matrix [24].

### 3.2. AFM

The surface structure of the films was evaluated using AFM (Figure 2II). The surface roughness of the films depended on the type of wax used. Among the AM-wax composite films, AM-CW exhibited the roughest morphology, with an arithmetic mean roughness (R_a_) of 186 nm and a root mean square roughness (R_q_) of 232 nm.

The AFM topography and phase images provided direct structural evidence for the formation of a wax surface layer. However, wax migration extent differed by wax type, owing to differences in their physicochemical properties. These properties influenced their interactions with the film-forming matrix and their behavior during emulsification and drying [31]. Unlike the other phase images, the phase image of the AM-SW film showed distinct heterogeneous phases (Figure 2(a_2_)). SW was discontinuously distributed on the film surface in a random sheet-like pattern, indicating that agar, maltodextrin, and SW formed a partially compatible system. The AFM observation of the AM-SW film was consistent with the SEM results. In contrast, PW, BW, CW, and CRW appeared to be distributed on the film surface in a more uniform manner. The AM-CRW film exhibited surface depressions and porous structures, which may correspond to the interstitial spaces formed in the three-dimensional film matrix network [32].

### 3.3. ATR-FTIR Analysis

ATR-FTIR analysis (Figure 4) was performed to investigate the intermolecular interactions at the upper (air-side) and lower (plate-contact-side) surfaces of agar/maltodextrin (AM) films incorporated with various waxes (SW, PW, BW, CW, and CRW). Compared to the infrared spectrum of pure wax (Figure 4c), no additional peaks appeared in the film spectrum, indicating that the films were merely a physical blend of the individual components, without the formation of new compounds through chemical reactions.

The FTIR spectrum showed a broad band near 3300 cm^−1^, corresponding to O-H stretching vibrations, and characteristic absorption peaks around 1019 cm^−1^, which were attributed to the C-O stretching vibrations of the 3,6-anhydro-galactose and anhydroglucose units present in agar and maltodextrin, respectively [33]. The peaks observed at 2916 cm^−1^ and 2849 cm^−1^ were primarily due to the CH_2_ asymmetric and symmetric stretching vibrations of the waxes, while the peak at 1464 cm^−1^ was assigned to the CH_2_ bending vibrations [34,35]. The peak observed at 719 cm^−1^ resulted from the rocking vibrations of the -CH_2_ and -CH_3_ groups [36]. The incorporated waxes exhibited distinct carbonyl characteristics, with a peak around 1740 cm^−1^ attributed to C=O stretching vibrations in wax esters [37], and a sharp peak at 1173 cm^−1^, which corresponded to C–O–C bond stretching, typically associated with esters [38]. The absence of these two peaks in paraffin wax confirmed the lack of ester groups in paraffin wax (Figure 4c).

Compared to the lower surface (Figure 4b), the upper surface (Figure 4a) exhibited broader and weaker O-H and C-O absorption bands associated with the polysaccharide matrix but stronger and sharper C-H and C=O peak intensities originating from the wax components. This phenomenon was attributed to the tendency of wax components to aggregate and migrate toward the upper surface during film formation, resulting in a wax-enriched upper layer above a compact AM network. In contrast, the lower surface spectra displayed an inverse trend in the intensities of these characteristic peaks. The upper surface spectra (Figure 4a) of the films showed a remarkable similarity to those of pure wax (Figure 4c), particularly evident in the AM-CW film, which further supported the formation of a wax-rich surface layer.

The O-H peaks associated with the AM matrix in the upper surface spectra shifted to higher wavenumbers compared to those in the lower surface (around 3300 cm^−1^), suggesting that wax incorporation affected the upper network by weakening the hydrogen bonds. Gao, Liu, Song, Huang, Dai, Hou and Wang [31] reported similar findings by incorporating different types of natural waxes into starch films. The AM-CW film exhibited a pronounced blue shift to 3363 cm^−1^, indicating that candelilla wax incorporation caused a greater disruption of hydrogen bonding in the upper matrix. Likewise, the absorption bands around 1019 cm^−1^ associated with C-O stretching of the agar and maltodextrin shifted to higher wavenumbers in the upper surface spectra, further indicating weaker interactions between components in the upper layer [39].

In the FTIR spectra of the upper surface of the AM-SW film, the intensities of the characteristic peaks at 2916 cm^−1^ and 2849 cm^−1^ were significantly lower than those of the other films. This may be attributed to the structural composition of soy wax differing from that of other waxes, which is supported by the FTIR spectrum of pure soy wax (Figure 4c). Similar observations were reported by Jahangiri, Mohanty, Pal, Clemmer, Gregori and Misra [38].

### 3.4. Thermal Properties

Differential scanning calorimetry (DSC) was performed to investigate the phase separation behavior potentially present in different film networks [40]. As shown in Figure 5a, the DSC curves of AM-wax films exhibited two distinct endothermic peaks, indicating phase separation. This behavior suggests that the hydrophobic wax phase crystallizes independently within the hydrophilic phase. The lower-temperature peak corresponded to the melting of the wax component, whereas the higher-temperature peak was attributed to the melting of the agar/maltodextrin matrix.

According to Figure 5b, each pure wax sample (SW, PW, BW, CW, and CRW) exhibited a distinct melting peak at approximately 49.9, 61.4, 64.7, 70.6, and 80.4 °C, respectively, matching their characteristic melting points. However, the addition of these waxes to AM films lowered the melting temperatures of the wax phase compared with pure waxes, which indicated that interactions between wax and AM components altered the polymer network structure. As the melting point of the added wax increased, the endothermic peaks of the AM matrix shifted to a higher temperature and then to a lower temperature. Notably, the AM-BW film displayed the highest AM melting temperature, indicating the greatest AM thermal stability compared to all other films. These thermal variations suggest a phase transition mechanism dependent on both the wax melting points and their spatial distribution within the film matrix. Waxes with melting points below the drying temperature can adequately spread to form continuous wax layers upon drying, which facilitates a compact AM network structure. The formation of such strong and compact network structures improves thermal stability [41]. Conversely, the higher melting point of CRW limits its flow and coalescence; consequently, the remaining solid CRW domains introduce structural discontinuities into the continuous AM matrix, leading to a decrease in AM thermal stability.

The maximum ΔH values for the characteristic peaks of wax and AM were observed in the AM-BW film (Table 1). This finding implies that the AM-BW film formed a two-phase system characterized by significant phase separation, resulting in a more compact and ordered AM microstructure. The AM-SW film exhibited the lowest ΔH_wax_ value, which was mainly attributed to the intrinsic characteristics of SW. Surendran, Ajjarapu, Arumugham, Kate and Satyavolu [30] demonstrated that the fusion enthalpy of SW is relatively low, indicating that less energy is required to break the bonds between the SW molecules and components.

### 3.5. XRD Analysis

The XRD patterns and crystallinity of AM-wax films are presented in Figure 6a and Table 1, respectively. The diffraction peak around 2θ = 19.3° resulted from the combined contributions of agar and maltodextrin [42,43]. The shoulder peak at 2θ = 13.8°, associated with the locally ordered structure of agar in the AM film [29], was absent in all AM-wax films, suggesting a transition to a partially amorphous state for agar. This absence is largely attributed to the high drying temperature, which affected the agar by weakening the constraints of the polymer network. Furthermore, the diffraction peaks at 2θ = 21.3° and 23.6° indicated that the wax crystallized in an orthorhombic form [44].

The incorporation of different waxes influenced the crystalline behavior of the polysaccharide matrix. As shown in Figure 6a, all composite films, except AM-SW and AM-CRW, displayed broader and less intense peaks at 2θ = 19.3° (especially for AM-PW film), suggesting enhanced compatibility between agar and maltodextrin. Thakur et al. [45] indicated that the interactions, which caused the peak broadening, serve as good indicators of the miscibility between the film ingredients. The intensities of the wax-related diffraction peaks at 2θ = 21.3° and 23.6°, along with the crystallinity values, exhibited an increasing trend with increasing wax melting points.

The crystallinity values of AM-SW and AM-PW remained at a similarly low level without significant statistical difference, whereas AM-CW and AM-CRW exhibited significantly higher crystallinity (Table 1). Films incorporated with lower-melting-point waxes (AM-SW) exhibited the weakest wax-related peak intensities and similarly low crystallinity values. Surendran, Ajjarapu, Arumugham, Kate and Satyavolu [30] observed that SW was amorphous in nature, as evidenced by its XRD pattern, which resulted in low crystallinity in the samples. Furthermore, the intensities of the AM-related diffraction peaks in the AM-PW film were notably weaker compared to those in the other films, which is likely responsible for their lower overall crystallinity. In comparison, the AM-CRW film exhibited the most intense wax-related diffraction peaks and a substantially higher crystallinity, indicating a more pronounced crystalline structure, despite partial CRW being randomly crystallized within the AM matrix. Cheng, Zhai, Wu, Li, Zhang, Sun, Wang and Hou [18] reported that CRW had a higher viscosity than BW and CW, which made it harder for CRW to break into small droplets during the film-forming process and resulted in larger wax particles in the film. Xiao, Wang, Wang, Liu, Liu, Zhang and Zhao [19] also indicated that a lower-viscosity palm oil was readily broken into smaller droplets under high-speed shearing during film preparation. The amorphous characteristics of low-melting-point waxes limit the film crystallinity, while high-melting-point waxes provide more stable crystalline phases.

Beyond the inherent structural characteristics of the waxes, film crystallinity is also influenced by the wax distribution. The aggregation of wax droplets into a continuous layer enhances the crystalline structure. This morphological dependence aligns with previous research indicating that larger particle sizes favor higher crystallinity in the lipid phase [46], thereby explaining why the crystallinity of the AM-CW film is comparable to that of the AM-CRW film.

### 3.6. Raman Spectroscopy

Raman spectroscopy was employed as an analytical method to investigate the molecular structures. The Raman spectra of the AM-wax films are presented in Figure 6b. The samples exhibited similar characteristic bands, suggesting the presence of comparable chemical compositions across the films. The Raman features at 1068 and 1135 cm^−1^ are due to C–C stretching vibrations. The peaks at 1301 cm^−1^ and 1444 cm^−1^ are assigned to the twisting and scissoring vibrational modes of the CH_2_ groups [47]. The characteristic absorptions at 1611 cm^−1^ and 1674 cm^−1^ in the AM-CRW and AM-SW films are likely due to C=C stretching vibrations [48]. In contrast, films incorporating PW, BW, and CW exhibited little or no spectral intensity in the 1500–1700 cm^−1^ region, confirming the absence of C=C groups. The vibrational modes observed in these films were predominantly those of saturated CH_2_ groups and C–C–C chains.

The Raman peak near 2850 cm^−1^ is attributed to the symmetric methylene stretching (d^+^), while the feature at 2885 cm^−1^ corresponds to the antisymmetric methylene stretching (d^−^) [49]. The intensity ratio of the d^−^ to d^+^ modes serves as a widely used empirical marker for alkyl chain order, with d^−^/d^+^ values ranging from approximately 1.6 to 2.0 for highly ordered crystalline solids to less than 0.9 for disordered liquid alkyl chains [50]. Accordingly, all composite film samples, except AM-SW, exhibited ratios exceeding 1.6, confirming that the alkyl chains in these films were predominantly crystalline.

### 3.7. Optical Properties

Across the 400–800 nm wavelength range, film light transmittance increased gradually with rising wavelength (Figure 4d). Film transmittance also increased when the melting point of the incorporated wax decreased. High transparency of emulsion-based films is closely associated with uniform film structure [51]. Among these, the AM-CRW film showed the lowest transmittance in this spectral region. Such weak light permeability mainly resulted from the uneven distribution of solid CRW within the film matrix. According to Galus and Kadzińska [21], lipid droplets dispersed within the film matrix affected transparency by blocking light penetration.

Furthermore, the low transmittance of AM-CRW film was also attributed to its crystalline characteristics. The decline in transparency also stemmed from the addition of high-crystallinity waxes, which increased light reflection [19,31]. By contrast, the AM-SW film with lower crystallinity exhibited weaker light reflection and consequently higher transparency.

### 3.8. Water Barrier Properties

Water vapor barrier performance is a crucial parameter for assessing the suitability of edible films. A lower water vapor permeability (WVP) value indicates better water barrier performance. As shown in Figure 7a, the incorporation of different waxes affected the WVP of the films to varying extents.

The AM-CW film exhibited a significant reduction in WVP (*p* < 0.05), demonstrating the best water vapor barrier performance among the films. This result is consistent with previous studies, showing that candelilla wax provides effective moisture barrier properties, with a lower WVP than those of beeswax and carnauba wax films [52]. In addition to the type and concentration of lipids, barrier properties are also governed by factors such as viscoelasticity, lipid particle size, the interactions between lipids and polymers, and the distribution of the lipid phase within the film matrix [53]. Notably, AM-wax films with clear layered structures typically exhibited better water barrier performance. This aligns with investigations confirming that bilayer films are generally more effective barriers against water vapor transfer than emulsion films owing to the presence of a continuous hydrophobic phase [25]. The formation of a wax-rich layer on the surface compacted the network, providing a continuous low-polarity domain and conferring hydrophobic protection.

The AM-CRW film exhibited poor water vapor barrier performance compared with the other films. This was mainly due to the incomplete migration of CRW during film formation. Some CRW particles were randomly embedded within the AM matrix, which may have hindered intermolecular interactions, resulting in a looser network structure with enlarged molecular gaps and thus facilitating water vapor transmission. Similar findings were reported by Xiao et al. [54], who found that adding virgin coconut oil to the polysaccharide matrix created numerous interfaces, reducing film compactness and increasing free volume, which accelerated water molecule diffusion and raised WVP values.

The water vapor barrier properties of biopolymer films are also influenced by their structural characteristics. Kowalczyk and Baraniak [55] suggested that adding solid non-polar components up to a certain quantity might create more void spaces at the polymer-wax interface, thereby promoting water molecule transport within the film. Voids and cracks were seen in cross-sectional SEM images and schematic diagrams of the AM-SW and AM-BW films (Figure 2(A_3_,C_3_) and Figure 3), which could explain why these two films exhibited weaker water vapor barrier performance compared with the AM-CW film.

### 3.9. Mechanical Properties

Prior to evaluating the mechanical strength, the film thicknesses were measured and presented in Table 1. The thickness of the AM-wax films varied significantly depending on the incorporated wax type (*p* < 0.05). Such variations can be attributed to the differing phase separation behaviors, wax migration extents, and lipid droplet sizes formed within the AM matrix during the drying process, which collectively altered the spatial volume of the resulting pseudo-bilayer film structures.

The mechanical properties of biodegradable films are typically associated with their network structures and intermolecular forces [56]. As the melting points of the incorporated waxes increased, the tensile strength of composite films was markedly improved (*p* < 0.05). This could be attributed to the high crystallinity of the films. Previous studies have also shown a strong correlation between the tensile strength of films and their crystallinity [57,58]. The AM-CRW film achieved the highest TS (22.57 MPa) and YM (867.99 MPa). The addition of CRW to the film matrix can improve TS, which is consistent with findings by Cheng, Zhai, Wu, Li, Zhang, Sun, Wang and Hou [18]. Chiumarelli and Hubinger [59] also reported that films with higher CRW content showed higher stress values, forming a more rigid polymer matrix with the highest elastic modulus.

Except for the AM-BW film, EAB increased as the wax melting points decreased (Figure 7b). The EAB values are closely affected by the physical state of wax within the film matrix. During film stretching, lipid droplets in films readily deform in a liquid state, whereas such deformation does not occur in a solid state [60]. With its lower melting point, SW remains relatively soft and partially plastic at ambient temperature, thereby exerting a plasticizing effect on the film. Nassef et al. [61] demonstrated that the chemical structure of SW results in a more elastic and consistent structure of fatty acids and hydrocarbons. On the contrary, CRW, the hardest natural wax with the highest melting point among commercial natural waxes [62], forms a more rigid dispersed phase in the film, reducing its ability to stretch and limiting the elongation of the AM-CRW film to merely 3.59%. Interestingly, the AM-BW film displayed a simultaneous increase in both TS and EAB, achieving the highest elongation value (23.55%) among all prepared films. Similar results were reported by Cheng et al. [63], in which beeswax incorporation improved both tensile strength and elongation at break of starch/gelatin films. This might be explained by the plasticizing effect of beeswax, which enhanced the flexibility of the film polymer chains, thereby increasing their mobility [64].

### 3.10. Dynamic Contact Angle

The dynamic contact angles of AM-Wax films measured over 150 s are shown in Figure 8a. All composite films demonstrated relatively high initial contact angles exceeding 86°, sharply contrasting with the initial values of 43° recorded for the AM film prepared in our previous study [29]. This observation suggests that wax incorporation notably enhanced the surface hydrophobicity of the AM film.

As the melting points of the added waxes rose, dynamic contact angles first increased and then decreased. Among the composite films, the AM-CW film presented the highest initial contact angle, while the AM-BW film achieved the maximum final contact angle. In comparison, the AM-CRW film exhibited the lowest contact angle, which decreased from 86.54° to 82.69° throughout the measurement period. These findings indicate that CW and BW droplets underwent more complete phase separation with the polysaccharide matrix during film formation. This phase separation, along with wax migration, aggregation, and surface recrystallization, improved film surface hydrophobicity. In contrast, the high melting point of CRW led to rapid solidification during drying, causing random crystallization of CRW within the AM matrix. The limited distribution of CRW crystals on the film surface made its hydrophobicity lower than that of other wax-incorporated films. Additionally, wax hydrophobicity is influenced by varying proportions of its chemical constituents, including hydrocarbons, alcohols, fatty acids, and wax esters [18].

### 3.11. Surface Energy

Surface energy is defined as the excess energy at the surface of a material compared to its bulk; that is, it represents the work required to create a unit surface area [65]. The surface energy of a material, whether solid or liquid, arises from both dispersive and polar interactions between molecules. Materials with low surface energy generally exhibit pronounced hydrophobic and oleophobic properties.

Figure 8b presents the measured diiodomethane contact angle, dispersive component, polar component, and total surface energy for the AM-wax films. Water and diiodomethane contact angles initially increased and then decreased with increasing wax melting point, with the AM-CW film showing the highest values. Surface energy followed the opposite trend, indicating that the AM-CW film surface exhibited both hydrophobic and oleophobic characteristics. This phenomenon arises from both the inherent hydrophobic nature of the wax and the formation of a continuous hydrophobic wax layer on the film surface.

## 4. Conclusions

This study elucidates how phase separation dictates lipid spatial distribution, crystallization, and structure–performance relationships in agar/maltodextrin (AM)-based pseudo-bilayer films. The intrinsic melting points of the incorporated lipid phase govern the competition between buoyancy-driven lipid migration and the solidification of AM matrix, thereby dictating the final spatial distribution of lipids within the film. An optimal melting point (e.g., as in candelilla wax) ensures sufficient fluidity during the drying process, enabling complete interfacial migration before matrix solidification. This forms a well-defined pseudo-bilayer structure, optimizing surface hydrophobicity and water vapor barrier performance. Conversely, mismatched melting points cause loose layer formation driven by low crystallinity (e.g., soy wax), or premature crystallization (e.g., carnauba wax) that disrupts matrix continuity. Independent of stratification, higher melting points of the incorporated waxes consistently increase film crystallinity, alkyl chain ordering, and tensile strength. Controlling lipid spatial distribution via phase separation is a viable strategy for designing biodegradable packaging. For future research, the scalability of this emulsion-casting process and the structural stability of the resulting pseudo-bilayer films under long-term storage should be systematically evaluated.

## Figures and Tables

**Figure 1:**
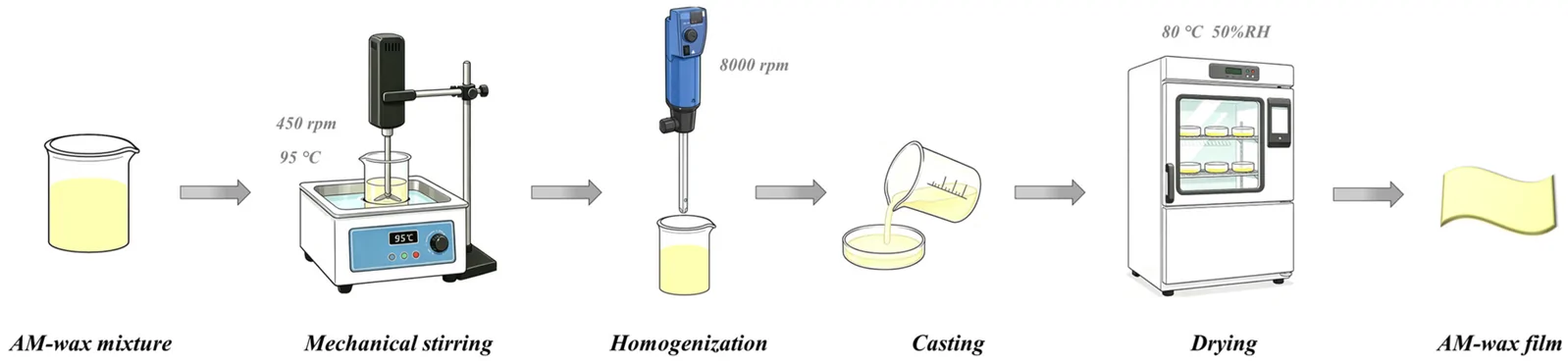
Schematic for the preparation process of AM-wax films.

**Figure 2:**
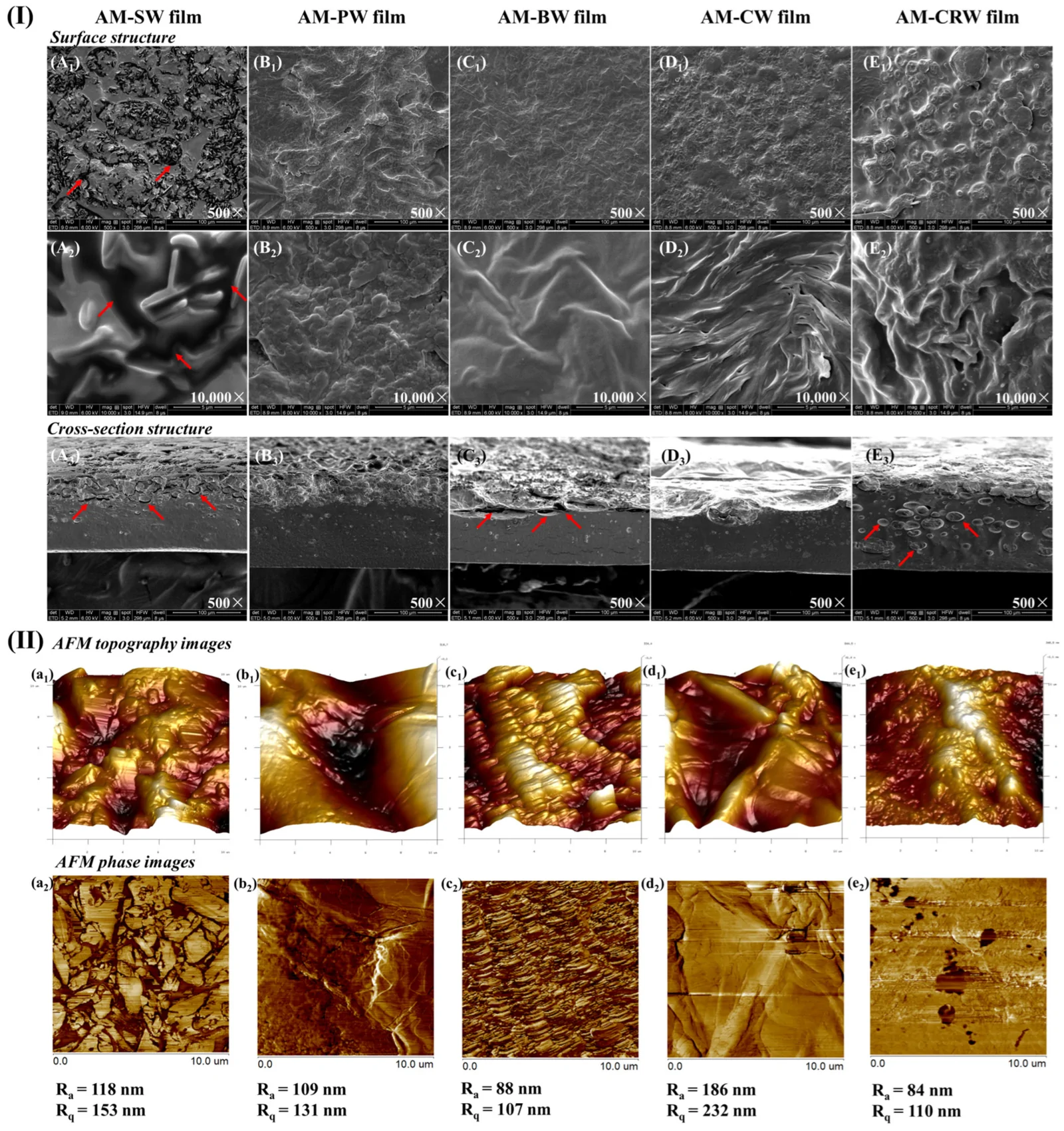
(**I**) SEM images of the upper surfaces (**A_1_**–**E_2_**) and cross-sections (**A_3_**–**E_3_**) of AM-wax films; (**II**) AFM topography (**a_1_**–**e_1_**) and phase images (**a_2_**–**e_2_**) of AM-wax films. Abbreviations: AM, agar/maltodextrin; SW, soy wax; PW, paraffin wax; BW, beeswax; CW, candelilla wax; CRW, carnauba wax.

**Figure 3:**
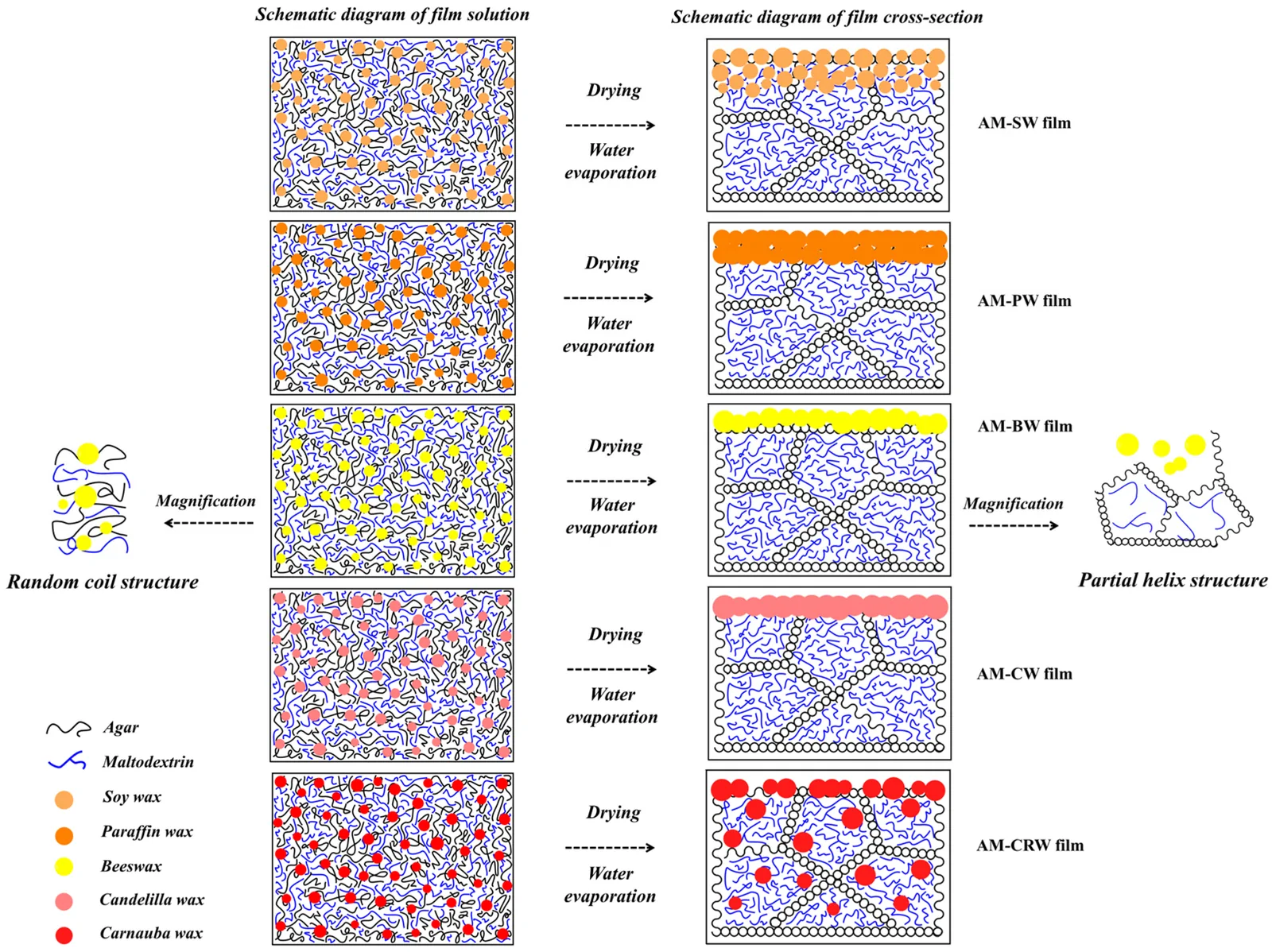
Schematic diagrams of the film-forming solution and film cross-sectional structure of AM-wax films. Abbreviations: AM, agar/maltodextrin; SW, soy wax; PW, paraffin wax; BW, beeswax; CW, candelilla wax; CRW, carnauba wax.

**Figure 4:**
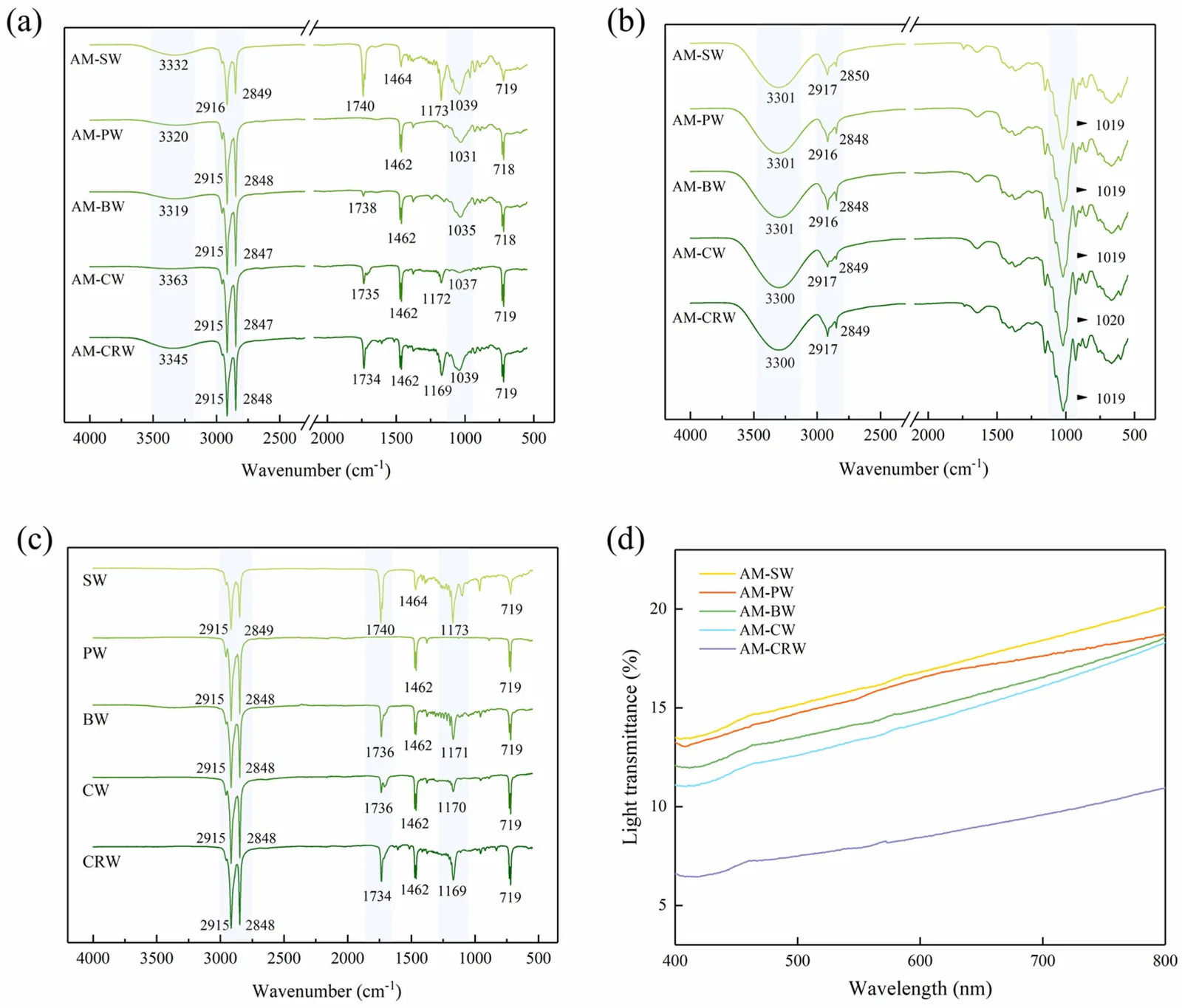
ATR-FTIR spectra of AM-wax films ((**a**): upper surface, (**b**): lower surface) and (**c**) pure wax components, and (**d**) optical properties of AM-wax films. Abbreviations: AM, agar/maltodextrin; SW, soy wax; PW, paraffin wax; BW, beeswax; CW, candelilla wax; CRW, carnauba wax.

**Figure 5:**
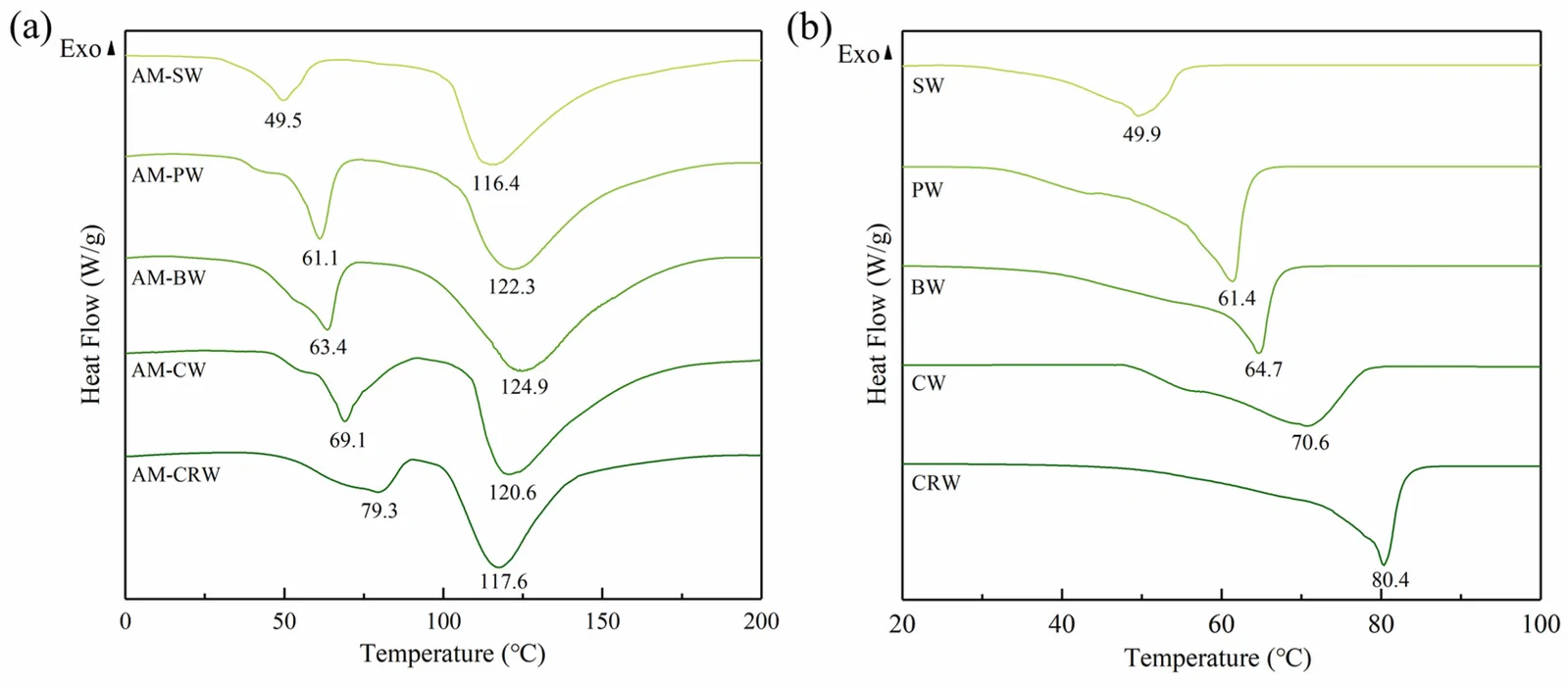
DSC thermograms of (**a**) AM-wax films and (**b**) pure wax components. Abbreviations: AM, agar/maltodextrin; SW, soy wax; PW, paraffin wax; BW, beeswax; CW, candelilla wax; CRW, carnauba wax.

**Figure 6:**
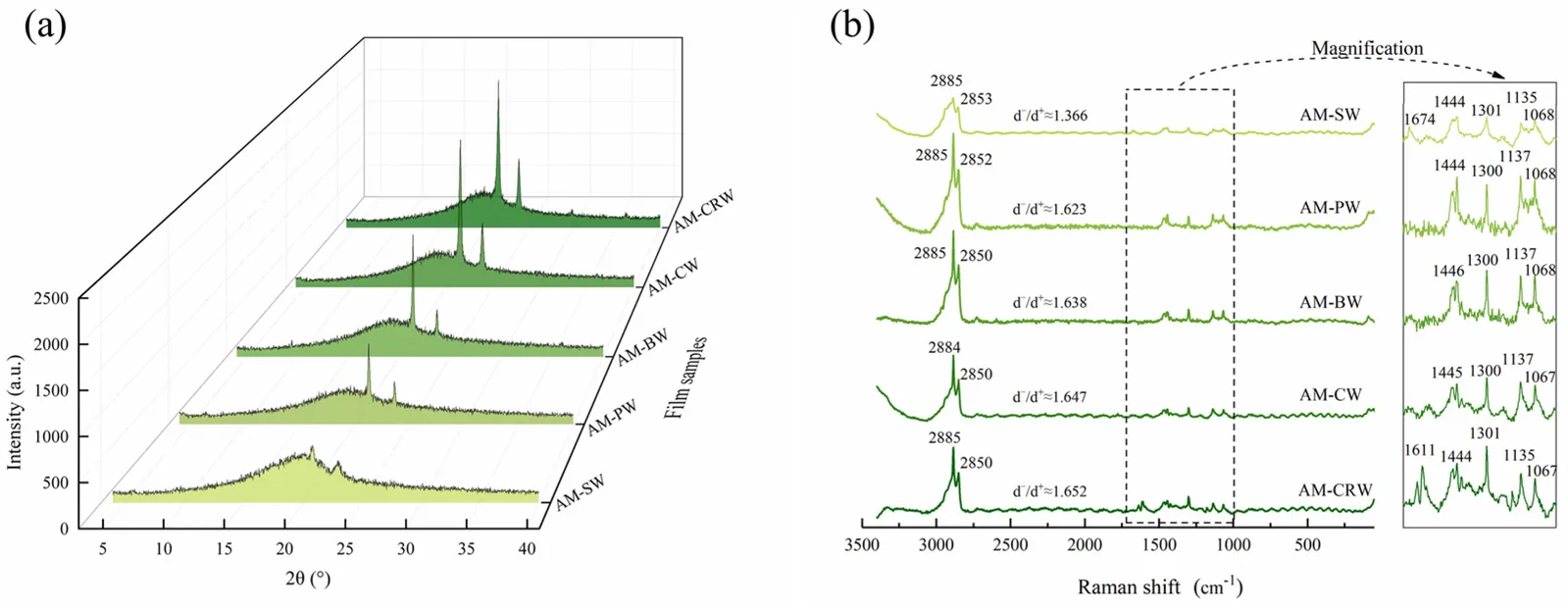
XRD patterns (**a**) and Raman spectra (**b**) of the AM-wax films. Abbreviations: AM, agar/maltodextrin; SW, soy wax; PW, paraffin wax; BW, beeswax; CW, candelilla wax; CRW, carnauba wax.

**Figure 7:**
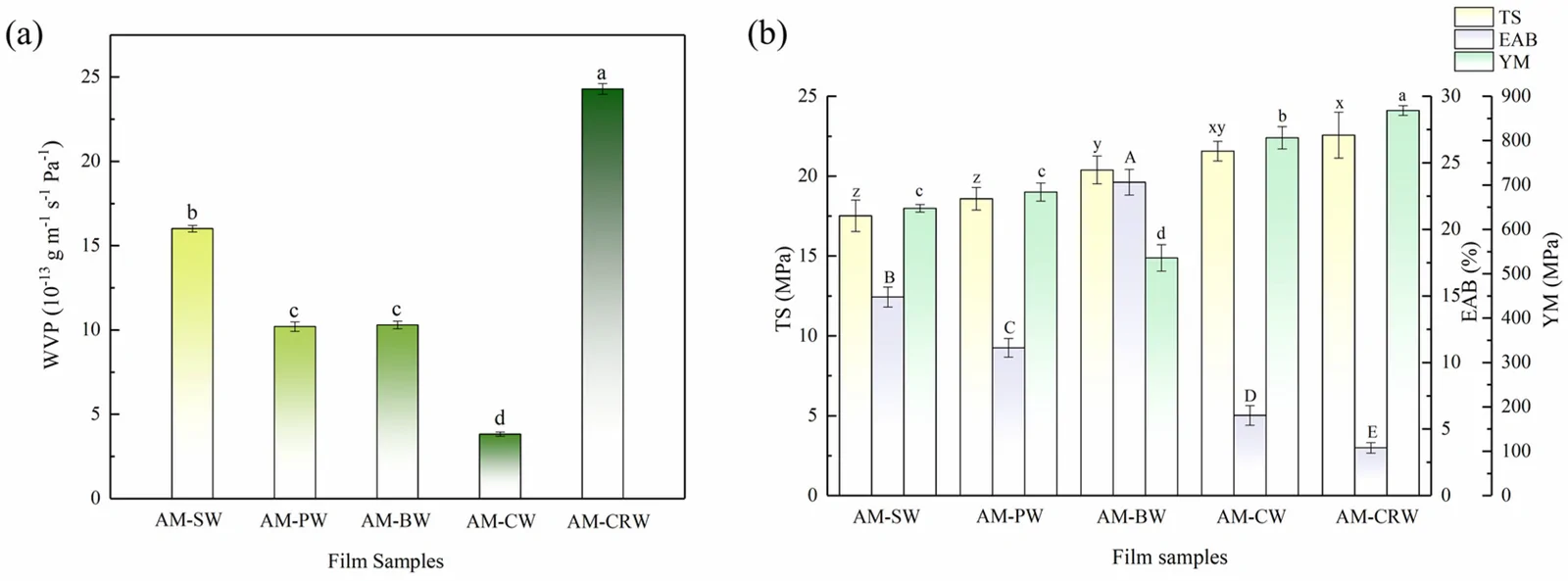
Water barrier properties (**a**) and mechanical properties (**b**) of the AM-wax films. ^a–d^, ^A–E^, and ^x–z^: Different superscript letters mean significant differences at *p* < 0.05. Abbreviations: AM, agar/maltodextrin; SW, soy wax; PW, paraffin wax; BW, beeswax; CW, candelilla wax; CRW, carnauba wax.

**Figure 8:**
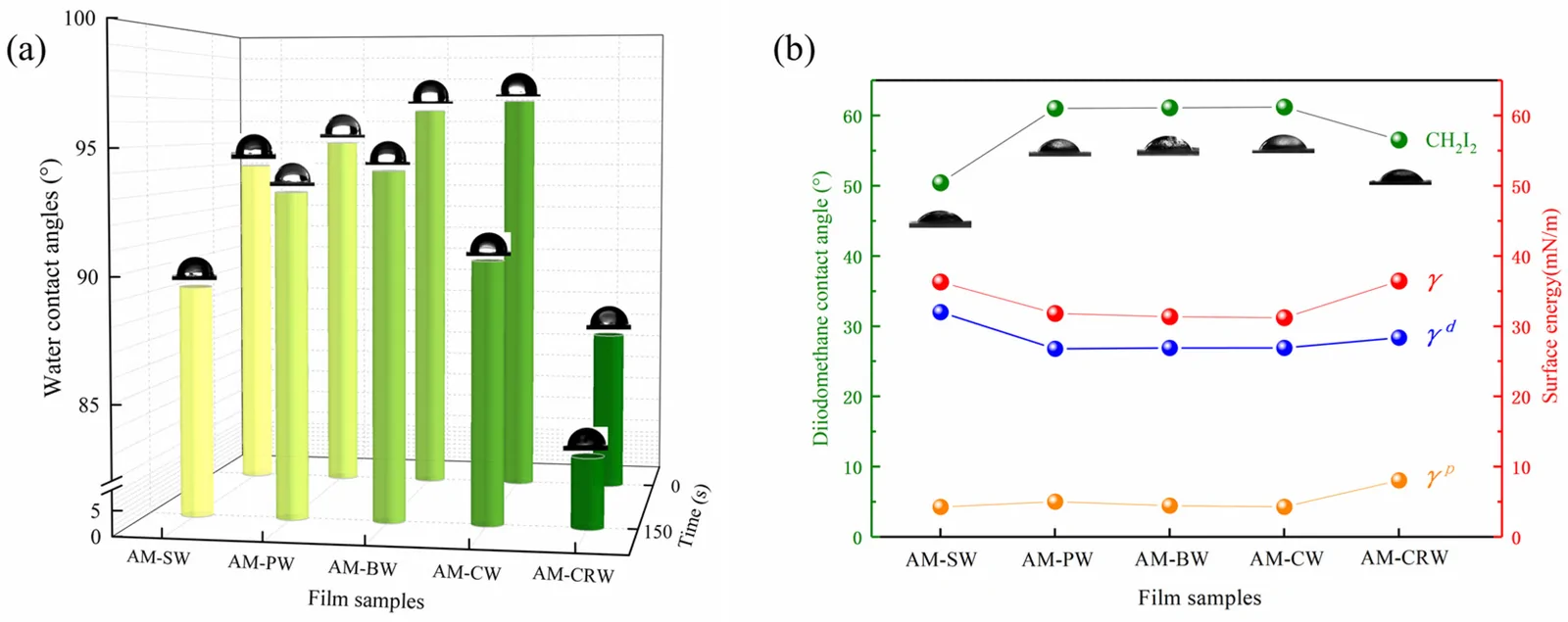
Dynamic contact angles (**a**) and surface energy (**b**) of the AM-wax films. Abbreviations: AM, agar/maltodextrin; SW, soy wax; PW, paraffin wax; BW, beeswax; CW, candelilla wax; CRW, carnauba wax.

**Table 1:** Enthalpy changes, crystallinity, and thickness of AM-wax films.

Film Samples	ΔH_wax_ (J/g)	ΔH_AM_ (J/g)	Crystallinity (%)	Thickness (μm)
AM-SW	19.34 ± 1.17 ^d^	94.66 ± 1.30 ^d^	18.71 ± 0.33 ^c^	112.67 ± 1.53 ^c^
AM-PW	28.73 ± 0.64 ^b^	111.10 ± 1.98 ^b^	19.29 ± 0.31 ^c^	138.00 ± 1.00 ^a^
AM-BW	33.99 ± 0.72 ^a^	116.04 ± 0.63 ^a^	23.71 ± 0.16 ^b^	95.67 ± 2.52 ^e^
AM-CW	32.93 ± 0.83 ^a^	110.53 ± 1.60 ^b^	25.19 ± 0.34 ^a^	108.33 ± 1.53 ^d^
AM-CRW	25.67 ± 0.64 ^c^	103.24 ± 1.46 ^c^	25.76 ± 0.40 ^a^	132.33 ± 2.08 ^b^

^a–e^ Different superscript letters in the same column indicate significant differences at *p* < 0.05.

## Data Availability

The original contributions presented in this study are included in the article. Further inquiries can be directed to the corresponding authors.
